# Prescriber perceptions of the safety and efficacy of unfractionated heparin versus low molecular weight heparin in the acute treatment phase: a qualitative study

**DOI:** 10.1080/20523211.2024.2418367

**Published:** 2024-11-19

**Authors:** Danielle Green, Catherine Edmunds, Roselyn Rose’Meyer, Indu Singh, H. Laetitia Hattingh

**Affiliations:** a Pharmacy Department, Gold Coast Hospital and Health Service, Southport, Australia; b School of Pharmacy and Medical Sciences, Griffith University, Southport, Australia; c Allied Health Research, Gold Coast Health, Southport, Australia; d School of Pharmacy, The University of Queensland, St Lucia, Australia

**Keywords:** Intravenous unfractionated heparin, low molecular weight heparin, anticoagulation, venous thromboembolism, acute coronary syndrome, stroke

## Abstract

**Background:**

Intravenous unfractionated heparin (IVUFH) and low molecular weight heparins (LMWH) are first line anticoagulants for the management of acutely unwell patients. The decision to prescribe either IVUFH or an LMWH is complex with minimal direction from clinical guidelines. The aim of this study was to explore individual prescribers’ perceptions on prescribing IVUFH or LMWH in patients’ acute management.

**Methods:**

Semi-structured interviews were conducted with purposively selected senior medical officers who were from specialities including cardiology, cardiothoracic surgery, respiratory, emergency, vascular surgery, nephrology, neurology and general medicine, identified as those that routinely prescribe IVUFH or LMWH. An interview tool with seven questions and four hypothetical case scenarios guided interview discussions. Interviews were audio recorded, transcribed and inductively coded for thematic analysis.

**Results:**

Twelve doctors participated in interviews between February and October 2022. Mean interview duration was 24 min; data saturation was achieved. Most were senior doctors: one was a registrar and others were staff specialists. Three key themes emerged: (1) rationale for the choice of heparinoid, (2) patient safety considerations and (3) resources required. The themes and subthemes identified the complexity of issues to consider when choosing between IVUFH and LMWH. Multiple factors were considered by participants which were based on previous experiences and institutional capabilities rather than evidence-based medicine.

**Conclusion:**

Future interventions should focus on highlighting LMWH as the preferred heparinoid in most clinical scenarios. The use of IVUFH should be reserved for specific patient cohorts where the benefit of IVUFH outweighs the additional risks.

## Background

Anticoagulants are the mainstay of therapy for patients who require rapid anticoagulation for life-threatening conditions (Smythe et al., 2016). Parenteral anticoagulation with a heparinoid is frequently used in acute care in patients with higher risk of deterioration or need for surgical intervention, where treatment with oral anticoagulation is not practical. The main parenteral agents used are unfractionated heparin (UFH) or low molecular weight heparin LMWH (Hull et al., 2023). For therapeutic anticoagulation, UFH is often administered as an initial intravenous bolus followed by an ongoing variable rate intravenous infusion (IVUFH). IVUFH requires complex prescribing algorithms, ongoing monitoring and dose adjustments, and intricate administration requirements. Anticoagulant effects of IVUFH are measured through laboratory tests with activated partial thromboplastic time (aPTT) the most widely used assay, requiring unlimited laboratory access and serial specimens, potentially every 4–6 h. IVUFH remains popular due to its efficacy, short half-life and the ability to be easily reversed with protamine, an intravenous antidote to neutralise the effects of heparins (Hull et al., 2023; Onwordi et al., 2018).

LMWHs, such as enoxaparin, are UFH derivatives with more predictable pharmacokinetics (Therapeutic Guidelines, 2023). Enoxaparin is administered by subcutaneous injection once or twice a day, offers a simplistic weight-based dosing schedule, has a stable and predictable patient response and does not routinely require therapeutic monitoring. If required, it can be measured via anti-Xa assay peak levels (4 h post-dose) prior to dosing. Enoxaparin has a longer onset and duration of action compared to IVUFH, significant renal clearance and cannot be reversed in full. This limits its use in certain clinical settings, such as in patients with renal impairment, obesity or those scheduled for imminent interventions (Medication Services Queensland, 2022).

There is limited evidence that one heparinoid agent is more efficacious or reduces overall mortality (Dolovich et al., 2000; Magee et al., 2003; Robertson & Jones, 2017; Sandercock & Leong, 2017) although there is literature to support LMWH use over IVUFH in some clinical situations. A systematic review of 29 randomised controlled trials found LMWH was superior in acute venous thromboembolism (VTE): reduced initial thrombus size, the recurrence of thrombotic complications and occurrence of major haemorrhage (Robertson & Jones, 2017). There is evidence that LMWH reduces the risk of heparin-induced thrombocytopenia (HIT) (Junqueira et al., 2017). LMWH is preferred in acute pulmonary embolism (PE) due to a lower risk of major bleeding and HIT (Konstantinides et al., 2020). A Cochrane systematic review found LMWH superior in acute coronary syndromes (ACS): lower risk of proceeding myocardial infarction, revascularisation and thrombocytopenia (Magee et al., 2003). However, both LMWH and UFH had a similar risk of mortality, recurrent angina and major or minor bleeding (Magee et al., 2003). In acute ischaemic stroke, there is no difference in overall mortality, however LWMH has been shown to have a significant reduction in risk of deep vein thrombosis (DVT) post stroke (Sandercock & Leong, 2017).

Clinical guidelines differ or are not clear in providing a rationale for using one agent over the other, with many using these agents interchangeably. The Thrombosis and Haemostasis Society of Australia and New Zealand recommends oral anticoagulants or LMWH if oral anticoagulants are not suitable for the acute management of VTE (Tran et al., 2019). The Australian Heart Foundation recommends either IVUFH or enoxaparin in patients with ACS at intermediate or high risk of ischemic events and in patients proceeding to percutaneous coronary intervention (PCI) and enoxaparin in patients undergoing fibrinolysis (Chew et al., 2016). Some international guidelines recommend IVUFH for patients proceeding to PCI except in those with ST-elevation ACS, whereby enoxaparin is preferred (Antman et al., 2006; Collet et al., 2021; Murphy et al., 2007). In stroke patients with cardio embolism, or cerebral venous sinus thrombosis (CVST), either agent can be utilised (Afshari et al., 2015; Stroke Foundation, 2022). For PE, LMWH are recommended over IVUFH for initial anticoagulation unless the patient is hemodynamically unstable or receiving reperfusion therapy (Konstantinides et al., 2020).

Queensland Health guidelines for public hospitals in Queensland, Australia, recommend LMWH for the management of VTE, either agent in ACS and IVUFH in patients with severe renal impairment or increased bleeding risk (Medication Services Queensland, 2022). At Gold Coast Hospital and Health Service (GCHHS), a Queensland Health public health service, local guidelines support the use of LMWH in PE and either heparinoid in other indications. Due to the lack of decisive decision support tools, the choice of anticoagulant often defaults to individual prescribers’ preference and experience. Local challenges with the prescribing and administration of IVUFH and the impact on patient safety prompted the need to obtain insights into prescribers’ decision-making processes.

## Methods

Qualitative methodology through semi-structured interviews with hospital doctors was utilised. The study was approved by the GCHHS Human Research Ethics Committee on 15 November 2021, reference number: (LNR) HREC/2021/QGC/81327. The COREQ (COnsolidated criteria for REporting Qualitative research) guidelines were utilised as the framework to underpin the research (25).

### Aims and objectives

The aim of this study was to identify aspects that influence doctors’ decision making between prescribing LMWH or IVUFH in patients’ acute management.

Specific objectives were to explore:

1. Patient characteristics that influence decision making.
2. Prescriber perceptions that influence decision making.
3. The influence of resources and decision support tools, institutional capabilities or capacity.

### Study sites/settings

This study was conducted at the GCHHS. This incorporated both Gold Coast University Hospital and Robina Hospital. The first being a ∼750 bed tertiary hospital facility and the latter being a ∼403 bed major regional hospital facility.

### Participants

A local audit in 2021 of 120 patients that were prescribed IVUFH or treatment dose LMWH for acute care showed that most prescribing occurred from specialties including cardiology, cardiothoracic surgery, respiratory, emergency, vascular surgery, nephrology, neurology and general medicine. These specialties were used as a focus for participant recruitment. Haematologists were also approached due to their expertise in the area. Inclusion criteria were Registrar (a medical practitioner who is undertaking an accredited course of study leading to a higher medical qualification) or Senior Medical Officer who routinely prescribed LMWH or IVUFH for the acute management of adult inpatients.

Prescribers using LMWH or IVUFH for prophylactic or novel indications (e.g. extracorporeal membrane oxygenation, intraoperative interventional radiology procedures, heparinisation for routine dialysis), and paediatricians and intensivists were excluded.

### Recruitment

Purposively selected potential participants who were from the specialty groups identified through the local audit were invited either face to face, via email or telephone to participate in a 20–30 min interview. Interested prescribers were provided with a research participant information statement and written consent form. Taking into account studies exploring the number of interviews required to reach saturation and the purpose of the research, it was estimated that between 12 and 20 interviews would be required to reach data saturation (Guest et al., 2006).

### Data collection tools

A semi-structured interview tool was developed (see Supplemental material) considering the literature (Afshari et al., 2015; Antman et al., 2006; Chew et al., 2016; Medication Services Queensland, 2022; Queensland Health, 2020; Tran et al., 2019) and research team members’ expertise. The tool consisted of both pre-determined open-ended questions and the opportunity for the interviewer to explore emerging themes or responses further and adapt questions as conversations progressed. This allowed for an in-depth exploration of experiences and opinions. The questions were assessed for content and face validity by four GCHHS clinicians and feedback incorporated. The tool was subsequently trialled to assess flow, length of interview and for the researcher to develop impartial interview skills. The final interview tool contained 10 demographic questions and eight clinical questions: seven questions and four case-related scenarios to prompt participants about prescribing decisions (see Supplemental material).

### Data collection

Interviews were conducted by three pharmacist team members (DG, CE or LH), employees of GCHHS but not working in the specific specialty areas of potential participants at the time of the study. One interviewee was a clinician researcher who is an experienced interviewer and provided mentoring and training to the remaining two interviewees who were experienced senior clinical pharmacists. Interviews were conducted either in person or via MS Teams and recorded via MS Teams or digital voice recorder. Field notes were taken to improve the validity of emerging themes. Audio voice only recordings were sent to an independent transcription service for transcribing. All transcripts were quality checked and participants offered the opportunity to review their transcripts.

### Data analysis

Thematic analysis was used to identify the principal patterns, experiences and topics that emerged from the interviews. NVivo version R1.6.1 (QSR International Pty Ltd) was utilised to facilitate coding and analysis of data via inductive reasoning. Qualitative rigor was applied by following a consistent data collection and analysis process to achieve credibility, transferability, dependability and confirmability (Denzin & Lincoln, 2018). This included the interviewer reflecting on interviews before sending a summary of findings to team members after each interview for input. Field notes and contact summaries were used with interview transcripts during data analysis. Initial codes were generated by CE and agreed on in collaboration with LH. The coding of all participants was independently checked by DG. Any uncertainties were discussed with the study team until consensus was reached. Subthemes developed from the initial themes were used to develop the analytical framework.

## Results

Forty-two doctors were contacted and twelve were interviewed between February and October 2022 (Table 1). Saturation was reached, meaning no new concepts emerged. The mean interview time was 24 min (SD 5.47, range 12–31 min).

**Table 1.** Interview participants’ demographic information.

Demographic criteria	No. participants (*n* = 12)	
	*n*	percent
Gender		
Male	10	83
Female	2	17
Age group (years)		
21–30	1	8
31–40	4	33
41–50	4	33
51–60	1	8
61+	2	17
Country of birth		
Australia	5	42
Other	7	58
University where obtained medical registration		
Australian	5	42
International	5	42
Unknown	2	17
Years worked as a doctor		
1–5	0	0
5–10	1	8
10–20	7	58
>20	4	33
Years worked as a doctor within GCHHS^a^		
1–5	4	33
5–10	3	25
10–20	4	33
>20	1	8
Specialist training		
General medicine	1	1
Haematology	2	17
Emergency	1	8
Nephrology	2	17
Respiratory	2	17
Cardiology	2	17
Neurology	1	8
Nil	1	8

^a^ Gold Coast Hospital and Health Service.

### Thematic analysis

Eighteen subthemes were identified and these informed three key interlinked themes (Table 2): rationale for choice of heparin, patient safety considerations and resources required.

**Table 2.** Summary of key themes and subthemes.

Theme	Subtheme
1. Rationale for choice of heparin	a. Critical thinking process b. Lack of evidence c. Guided by indication d. Application of pharmacokinetics
2. Patient safety considerations	a. Antiplatelet therapy b. Bleeding risk c. Haemodynamic stability d. Renal function e. Surgical intervention
3. Resources required	a. Administration complexity b. Alternative specialty input c. Bleeding risk d. Clinical experience e. Decision support tools f. Patients assigned inpatient unit g. Pharmacist support or recommendations h. Severity assessment i. Staff resources

### Rationale for choice of heparin

The choice between LMWH or IVUFH was identified as a challenging decision and participants explained what impacted decision-making processes. This theme incorporated four subthemes: critical thinking process, evidence, indication and pharmacokinetics, summarised in Table 3 with exemplar quotes.

**Table 3.** Exemplar quotes related to rationale for choice of heparin.

Key issues	Exemplar quotes
Critical thinking process	*You’re looking for reasons to deviate from giving them low molecular heparin, I think, is the way I probably think about it. It has to reach a sufficient threshold where I’m willing to accept the downsides of prescribing intravenous heparin, which are notable, and then it leads to further considerations as to how you mange those risks. It has to be individualised to the patient, because those two things are a dynamic assessment. – P3*
Lack of evidence	*There is no morbidity or mortality difference between the two from a prediction of thromboembolism perspective. – P10The literature basically shows, or says, there's no difference between either of them in terms of outcomes. – P6*
Guided by indication	*Each of those indications, I prefer to use enoxaparin because it's much more predictable, so it's less likely to produce inadequate protection or overdosing and risk of bleeding compared to unfractionated heparin. I think the only situation I would use it myself [IVUFH] is in people with the left main [coronary artery blockage], that's at the direction of the cardiothoracic surgeons. – P6*
Application of pharmacokinetics knowledge	*The disadvantage is that it's [IVUFH] intravenous and it's hard to get into the right therapeutic range. So sometimes you've got people you know on intravenous heparin, it's not actually doing anything or is dangerously high. The other advantage is that you can use it in any degree of renal impairment safely. It's also completely reversible. – P11There's probably half an hour later onset of action of enoxaparin compared to unfractionated heparin. But there's no mortality difference between using the two. So that doesn't translate to a clinically kind of useful parameter [to preferentially use IVUFH]. – P2*

#### A. Critical thinking process

A critical thinking process was followed by most participants when considering whether to prescribe an LMWH or IVUFH, described as a multifaceted, complex decision process with no clear criteria. Issues considered were the risk benefit ratio and if one agent would provide a safer outcome for the patient. It was noted that the more senior participants’ reasoning processes supported LMWH as a ‘first line default’.

#### B. Lack of evidence

Many participants agreed there is lack of evidence to suggest morbidity or mortality differences on thromboembolism outcomes of either agent when used in the acute treatment phase, however, many considered LMWH to have a superior safety profile.

#### C. Guided by indication

There was agreement that LMWH and IVUFH were reserved for specific indications. Participants agreed that they mostly prescribed therapeutic LMWH or IVUFH for common indications being DVT, PE, atrial fibrillation, warfarin replacement and ACS. Use in niche indications often involved consultation with literature and other specialists.

#### D. Application of pharmacokinetics knowledge

Participants provided detailed descriptions of their understanding of the pharmacokinetic differences such as dosing, onset of action, half-life and excretion between LMWH and IVUFH that impact their decision-making. IVUFH was acknowledged to have a faster onset and duration of action, however, this does not correlate to reaching therapeutic levels in a timely manner. The application of pharmacokinetic properties in the renally impaired or obese populations was a common consideration. Four participants raised the pharmacokinetic difficulties in dosing LMWH in obese patients, involving the need for dosing caps and using total body weight, lean body weight or ideal body weight to determine dose. Some participants suggested that IVUFH is a more appropriate choice in obese patients due to an initial total body weight capped dose, followed by measurable therapeutic levels thereafter, noting that routine blood sampling may be more difficult in morbidly obese patients.

Pharmacodynamic reversibility and availability of monitoring were also considered, with some noting limited availability of monitoring anti-Xa levels in a timely manner.

### Patient safety considerations

Patient safety was an important consideration and participants discussed key patient-specific factors that impacted decision making, categorised into five subthemes: antiplatelet therapy, bleeding risk, haemodynamic stability, renal function and surgical intervention. Exemplar quotes are summarised in Table 4.

**Table 4.** Exemplar quotes related to patient safety considerations.

Key issues	Exemplar quotes
Concomitant antiplatelet therapy	*There is one other group, and that is people who you’re concerned may be bleeding actively, and in whom is a compelling reason to give antiplatelet therapy, such as they’ve just had a stent put in. Until we find out whether they’re actively bleeding, they may go on to a heparin infusion, because it's something that can be turned off immediately. – P6*
Bleeding risk	*But I wouldn't say that bleeding risk is an absolute reason to choose UFH over low molecular weight, because it would be things like somebody that's – like, has just had an operation, or has recently had significant bleeding, or has had a recent intracranial haemorrhage, things like that. – P2*
Haemodynamic stability	*The main thing [to consider] is either the patient is unstable now or is, for whatever reason, based on clinical sort of information, you perceive them to be at risk of going from submassive to massive [pulmonary embolism] and requiring either systemic thrombolysis or interventional radiology techniques [favours IVUFH]. – P2*
Renal function	*The one group actually that I should mention that being an exception, the patients with significant renal impairment, because their enoxaparin will accumulate and will last longer, quite a bit longer. So probably they are better treated with an unfractionated heparin, but I can't remember the last time that that situation arose, actually. – P6*
Surgical intervention	*My understanding was that sometimes a speciality will request that [IVUFH]. Because if the patient goes to theatre or if they then have to revoke the stuff for whatever reason, it's easier to cease it. – P4*

#### A. Concomitant antiplatelet therapy

Two participants mentioned considering whether patients may already be on antiplatelet medications before choosing LMWH or IVUFH. Choice relied on if the patient is actively bleeding, at risk of bleeding and if there was a compelling reason to use antiplatelet medications in combination with anticoagulation therapy.

#### B. Bleeding risk

Most participants considered patients’ bleeding risk and ongoing safety before prescribing LWMH or IVUFH. Risk factors were taken into consideration *ad hoc* and included: haemoglobin level, recent surgery, previous major or minor bleeds, need for imminent thrombolysis, thrombocytopenia, history of bleeding, hypertension, age, renal function, other medications that affect bleeding risk and if there is an active bleed. Participants stated that those with a higher bleeding risk, either currently or in future, would be more likely to be prescribed IVUFH, due to the short half-life and the option to reverse it in full with protamine sulphate.

#### C. Haemodynamic stability

Participants agreed that IVUFH is a safer and more suitable alterative to LMWH in patients that are hemodynamically unstable due to the shorter half-life and ability to reverse IVUFH in full if required. There was no specific tool utilised or mentioned to assess haemodynamic stability.

#### D. Renal function

All participants acknowledged that LMWH should be used with caution in renal impairment. However, there was no consensus at what stage or degree of impairment IVUFH should be used with some participants referring to ‘significant’, ‘end-stage’ or ‘profound’ renal impairment. The majority indicated that an estimated glomerular filtration rate (eGFR) less than 30 mL/min contraindicates use of LMWH. A haematologist noted that nephrologists use LMWH in patients with severe renal impairment and both nephrologists stated they are comfortable using varying doses of LMWH in all stages of renal impairment. One nephrologist elaborated that there is, however, a period of concern where the patient has the potential to be inadequately anticoagulated whilst waiting for their 4th dose anti-Xa levels. This is due to the nature of chronic kidney disease (CKD) and the distinct interpatient variability in levels of residual renal function within individual stages of kidney disease.

#### E. Surgical intervention

Participants agreed that impending surgery was a consideration when choosing LMWH or IVUFH. However, it was not an absolute indication to use IVUFH. Considerations included the type of surgical intervention, surgeon preference as well as the time to surgery and thus potential for medication washout. It was noted that there was no consensus between surgeons or specific procedures and the state-wide protocol was not prescriptive and rather cumbersome to use. Participants agreed that the peri-operative choice of anticoagulant is not always evidence-based and, in some instances based on previous experiences. One participant reflected on a negative experience with IVUFH during a neurosurgical procedure and as consequence prefer enoxaparin.

### Resources required

Resourcing considerations were complex and multifaceted. These were categorised into nine subthemes: administration complexity, alternative specialty input, bleeding risk, clinical experience, decision support tools, patients’ assigned inpatient unit, pharmacist support or recommendations, severity assessment and staff resources. An overview of exemplar quotes is summarised in Table 5.

**Table 5.** Exemplar quotes related to resources required.

Key issues	Exemplar quotes
Administration complexity	*The [IVUFH] protocol is a disaster. So having to have your bloods checked every four hours, so the time for the junior doctors, it's just terrible. The time it takes, the faffing around and the poor patients being jabbed very four hours is just ridiculous. – P12*
Alternative speciality input	*Sorry if they're under us [haematology], we'd probably just handle it ourselves [choice of LMWH or IVUFH], but we wouldn't get involved, in anybody [other medical teams] other than if a patient that happened to have a cancer. – P11*
Bleeding risk	*[To assess bleeding risk] Generally, it will be based on clinical information. Some people will use the HAS-BLED score. I personally don't use that in the after-hours space, because most of these patients will have a higher HAS-BLED score and it actually does not preclude you from anticoagulation. Because the conditions that I mentioned, either you will have to anticoagulated, like in high-risk PE and stuff, or you're looking at a severe morbidity or mortality if you don't anticoagulate. – P10*
Support form experienced clinicians	*My personal thing is that for any process where you haven't had an experience in alleging a particular medication, if that the experience is not there, how do we support that person or clinician in that process is important. – P10*
Decision support tools	*I think the tool that I would suggest, and I know it's controversial, is a one-pager, with not too much written on it, to say what are the conditions in which you should consider using IV heparin?. – P10*
Patient's assigned inpatient unit	*I’m always very reticent to send acute PE [pulmonary embolism] on unfractionated heparin [IVUFH] to – just to a general Ward – P2*
Pharmacist support or recommendations	*So, if it happens [initiating IVUFH] during business hours, during work hours, then obviously the pharmacist on the ward will discuss with them these kind of scenarios, because obviously they will need to be aware of starting this and supporting the nursing team on the ward. But if it's after hours, then we have to initiate it, regardless of consultation with the pharmacist. – P10*
Severity assessment	*[in regards to pulmonary embolism] There's submassive where you're thinking about whether you should thrombolyse or send for catheter-directed thrombolysis or something like that, then for that you'd maybe go heparin but for the majority of them we'd still go Clexane. – P5*
Staff resources	*Most of the bed management issues are not under your control. So even if you were to say, alright, start this patient on this infusion because this nurse got a skill mix here, after-hours, you're not going to be guaranteed, because after eight hours another nurse is going to look after them. – P10*

#### A. Administration complexity

Many participants acknowledged that the complex nature of IVUFH deterred prescribing due to the varying and complex electronic prescribing nomograms, initial weight dependent bolus, four to six hourly aPTT monitoring, timely access to phlebotomy or nursing staff, variable infusion rates depending on aPTT results and interpatient variability to reach and sustain therapeutic range. Participants highlighted that even if the IVUFH nomogram is followed this does not mean that the patient will reach and maintain their aPTT in the therapeutic range. One explained that in their experience it takes over 24 h to reach a therapeutic level in most patients. The overall complexity of IVUFH was thought to increase the risk of medication-related harm.

#### B. Alternative speciality input

Participants were mostly confident in their own speciality to prescribe either IVUFH or LMWH although there were some instances where participants would seek alternative specialty input. These included potential for imminent surgery as the need to consult with the interventionist, or if malignant or haematological disorders were present haematology input would be requested. In the case of renal impairment or obesity, participants acknowledged the need to either dose adjust LMWH or utilise IVUFH, without frequently seeking nephrology or haematology advice. Participants acknowledged that all patients on IVUFH after hours must be referred to the After-Hours Care Unit to ensure continuity of haematological monitoring outside normal working hours.

#### C. Bleeding risk

Most participants acknowledged the lack of validated tools or resources to assess bleeding risk to guide appropriate anticoagulation. One participant used the HAS-BLED (Hwang, 2019) criteria to assess bleeding risk (scoring system to assess risk of major bleeding in those taking oral anticoagulants with atrial fibrillation). However, most of the participants did not use a specific resource or tool to assess risk but rather considered a patient's overall picture. It was noted that more experienced doctors described a more intuitive ‘holistic’ patient approach to assess bleeding risk without use of resources. However, participants stated that even in patients with a high bleeding risk, the urgent need to be anticoagulated often outweighed the risk of bleeding, thus rendering such tools irrelevant.

#### D. Support from experienced clinicians

Prior clinical experiences played an important role in decision making to choose IVUFH or LMWH. One participant acknowledged that a previous negative experience with IVUFH resulted in avoiding its use.

#### E. Decision support tools

Most participants stated they did not use decision support tools when deciding whether to prescribe IVUFH or LMWH. Participants acknowledged that there are specific prescriptive guidelines available for some clinical indications, however, these are often lengthy, cumbersome to access and only useful if recently updated. However, some mentioned using specific resources such as the Australian Medicines Handbook to determine renal function, Medical Calculators to assess bleeding risk and determine renal function, resources from another health service to adjust LMWH doses in obese patients and evidence-based clinical guidelines.

#### F. Patients’ assigned inpatient unit

The patient's inpatient unit allocation was a contributing factor when deciding on an anticoagulant. Participants suggested staff unfamiliarity with IVUFH was a significant issue that had potential to result in poor patient outcomes. They acknowledged that theoretically all inpatient units should have staff with skills to manage IVUFH although this was not the case in practice. Some went as far to say that IVUFH should only be used in an intensive care unit with others naming three to four inpatient units that they believed could manage IVUFH. One experienced doctor voiced frustration at having to consider patient's inpatient location when choosing to use IVUFH, suggesting that the choice of anticoagulant should be what is in the best clinical interest of the patient rather than the logistics of the inpatient unit.

#### G. Pharmacist support or recommendations

Some participants acknowledged the support an inpatient unit pharmacist provides when prescribing IVUFH or LWMH. This was explained as assistance to prescribe initially, ongoing monitoring and how to switch between anticoagulants. However, it was noted that pharmacist support was not always available, especially outside of business hours where junior medical officers are often more likely to need assistance.

#### H. Severity assessment

Three participants considered the severity of PE before selecting anticoagulation, with one mentioning use of a PE severity index to assess the risk (Jiménez et al., 2010). The consensus among all three participants was to use IVUFH in high-risk PE patients in combination with thrombolysis.

#### I. Staff resources

Staffing numbers, nursing experience and access to phlebotomy were all considerations prior to prescribing IVUFH. However, participants stated that bed management and staffing issues were within their control and at shift change these skills would not be guaranteed. It was acknowledged that as the frequency of prescribing IVUFH reduces, the knowledge and experience of staff is decreasing, and this will become more prevalent in the future. One participant mentioned that IVUFH is used more frequently in the United States and because of this he gained extensive experience. However, he explained that of his team of approximately 40 doctors, only 4–5 would have had exposure to prescribing IVUFH.

## Discussion

This qualitative study explored hospital doctors’ decision making when deciding to prescribe IVUFH or LMWH to manage high acuity patients. Thematic analysis of the data showed three main themes: (1) rationale for the choice of heparin, (2) patient safety considerations and (3) resources required. The themes and subthemes identified the complexity of issues to consider when choosing between IVUFH and LMWH. The lack of clear guidelines to support the choice of anticoagulant highlighted the need for future interventions to guide decision making.

The prescribing and administration of IVUFH requires prescribers and nursing staff to refer to lengthy medication guidelines (Medication Services Queensland, 2022) and local ‘quick reference guides’ for specific details for prescribing and administration within the electronic medical record system. Research showed that the standardisation of paper-based heparin management to a computerised protocol did little in the way of reducing medication errors with 2.01 IVUFH errors per 1000 doses when changing to an electronic medication record system (Leung & MacRae, 2019). This research incorporated a follow-up study at a Minneapolis hospital that showed subsequent improvements to the electronic IVUFH nomogram resulted in 11.4% reduction in errors during the first 3 months and a 37.8% reduction in errors in the following quarter.

Although participants acknowledged that LMWH should be used in caution with renal impairment there was no consensus as to what degree of renal impairment restricts its use and experienced nephrologists use LMWH in all stages of renal impairment. Many resources, including the Queensland Health state-wide guidelines, contraindicate LMWH with severe renal impairment (CrCl <30 mL/min) (Leung & MacRae, 2019; Medication Services Queensland, 2022), although there is growing evidence that LMWH can be used with reduced doses and potential for increased monitoring requirements (Nutescu et al., 2009). A meta-analysis of 18 studies that included non-dialysis dependent, renally impaired patients supports the use of empirically adjusted enoxaparin doses in renally impaired patients to reduce the bleeding risk associated with accumulation (Lim et al., 2006). The enoxaparin approved product information suggests a reduced treatment dose in severe renal impairment (1 mg/kg subcutaneously once daily) and although no dosage adjustment is recommended in patients with moderate and mild renal impairment, observation is advised for signs and symptoms of bleeding (MIMS Online [Internet], 2020). Other standard clinical references also recommend the reduced dose of 1 mg/kg once daily in severe renal impairment (Australian Medicines Handbook, 2023; Hull et al., 2023; Therapeutic Guidelines, 2023). Our data showed lack of prescribers’ awareness that LMWPs could be used in renally impaired patients. The conflicting evidence about the use of LMWH in patients with renal impairment highlights is a need for a clear consensus as to if and how LMWH could be utilised in severe renal impairment.

The appropriateness of LMWH in obesity was a common consideration, however, there was no consensus as to what body mass index (BMI) would warrant IVUFH rather than LMWH in obese patients (BMI ≥30 kg/m^2^). A retrospective audit conducted to measure the time taken to first reach a therapeutic aPTT with IVUFH that included 240 obese patients showed the time taken to reach therapeutic aPTT significantly increased as the patient's body weight increased (Shin & Harthan, 2015). Research also suggests that 29% of obese patients experience a delay of more than 24 h to reach therapeutic IVUFH levels (Ihaddadene & Carrier, 2016). Current available evidence in obesity suggests use of LMWH twice daily, dose adjusted on total body weight, not routinely capping dose (consider if BMI ≥ 40 kg/m^2^ or >150 mg dose required) and that assay monitoring is not routinely required (Ihaddadene & Carrier, 2016; McCaughan et al., 2018; Sebaaly & Covert, 2018). Anti-Xa monitoring may be required in cases where the elimination of LMWH is impaired, where there is an unexpected clinical response or to confirm compliance (Sebaaly & Covert, 2018). The use of LMWH dosed at actual body weight in obesity is also supported by QH state-wide guidelines, whereby initially enoxaparin dose should be 1 mg/kg twice daily with anti-Xa monitoring required if BMI > 40 kg/m^2^ or bodyweight greater than 120 kg (Medication Services Queensland, 2022). Our findings show a need for ongoing education supporting the use of LMWH in obese patients (McCaughan et al., 2018).

Participants considered various hospital resources including the skills and capability of nursing staff in inpatient units and pharmacists to review their prescribing and explained how there impacted their prescribing decisions. They acknowledged the absence of a single validated tool to assess bleeding risk across multiple indications. Most experienced participants used the gestalt approach, where a clinical assessment of individual risks is considered over application of decision support tools. A multinational, observational study of 15,156 medical patients was conducted to determine the in-hospital bleeding risk in acutely ill inpatients. A multiple regression model analysis identified risk factors at admission associated with bleeding outcomes which were used to develop a tool, the IMPROVE bleeding risk calculator, to determine a patient's bleeding risk at the point of hospital admission. A score ≥ 7 indicates an increased bleeding risk (44). The IMPROVE tool has since been validated and can be used to determine a patient's bleeding risk at the point of hospital admission (45, 46). The ability to rapidly and effectively reverse IVUFH cements its use as the preferred agent in those with a higher bleeding risk (Hull et al., 2023; Medication Services Queensland, 2022). The IMPROVE tool might be suitable to screen for patients whereby the increased risk of bleeding could flag a preference for IVUFH over LMWH or prompt for haematology option, depending on the patients clinical picture.

### Strengths and limitations

The research team consisted of clinicians with complementary skills and experience to facilitate meaningful discussions and interpretation of the data: two clinical researchers and university academics and three senior pharmacists working within governance, research and clinical roles. Participants were from one health service and the numbers limited to 12, however, saturation was reached. Cardiothoracic and vascular specialists were invited to participate but were not recruited. Medical officers were interviewed by pharmacists, this may have contributed to bias as medical officers were familiar with pharmacists within the health service, however, at the time of research no pharmacist was working closely with any participant. The LMWH used at the health service and referred to by participants was enoxaparin, therefore, findings from this study may not apply to other settings where different LMWH are used. However, the other LMWHs do have similar prescribing restrictions in patients with decreased renal function.

## Conclusion

The interviews highlighted that multiple factors are considered when deciding to prescribe IVUFH or LMWH. There was no agreement among participants regarding the preferred use of LMWH, indicating a need for clinical consensus to support the use of LMWH in obesity and multiple stages of renal impairment. Considering the evidence to support LMWH's improved safety, administration and in some instances morbidity or mortality outcomes, policy makers should consider updating procedures to support the preferential use of LMWH. In addition, it should be clarified under what circumstances IVUFH should be considered.

## Supplementary Material

UFH and LMWH INTERVIEW TOOL.docx

COREQ_checklist.docx
