# Supplementary material for: Prescriber perceptions of the safety and efficacy of unfractionated heparin versus low molecular weight heparin in the acute treatment phase: a qualitative study
Source: J Pharm Policy Pract. 2024 Nov 19;17(1):2418367. doi: 10.1080/20523211.2024.2418367 (PMC11578413; doi:10.1080/20523211.2024.2418367)
Supplement: UFH and LMWH INTERVIEW TOOL.docx [file JPPP_A_2418367_SM2593.docx]

**The safety and efficacy of unfractionated heparin in the acute treatment phase compared with low molecular weight heparin**

**Semi-Structured Interview Questions**

**Opening Remarks:**

**“Thank you for your time.**

I would like to start off with a brief introduction of myself: My name is __________________________ and I am [describe role]

The purpose of this interview is to explore aspects that influence doctors’ practices and decision-making when choosing between unfractionated heparin and low molecular weight heparin for anticoagulation in the acute treatment phase. This will help us in understanding decision-making processes to develop future interventions.

We really value your input and honest contribution. Anything you tell us will be treated with strictest confidence and your identity will be protected.

If you have any questions regarding this research, please do not hesitate to contact any of the research team members.

**The interview should take about 30 minutes.**

**--------------------------------------------------------------------------------------------------------------------------**

**Pre-interview information to be collected:**

1. Gender: 🞎 Male 🞎 Female 🞎 Other

2. Age group (years): 🞎 21-30 🞎 31-40 🞎 41-50 🞎 51-60 🞎 ≥ 61

3. Country/region of birth: 🞎 Australia 🞎 China 🞎 Malaysia 🞎 New Zealand

🞎 United Kingdom 🞎 Other, please specify _______________

4. At which university did you obtain your initial medical qualification?

______________________________________________________________________________

5. Which year did you graduate with your medical degree? __________________

6. In which year did you first obtain your registration to practise as a doctor in Australia?

7. How many years have you worked as a doctor? __________________

8. How long have you worked as a doctor at GCHHS? (months or years) __________________

9. If you have any specialist training, please list these __________________________________________

10. When did you obtain your specialist training? ____

**Before we begin, I would like to learn more about you.**

1. Can you please provide a brief outline of your current position at XX Health and what this involves?

- How long have you worked in this position?
- What are your current roles? If you have multiple roles, please specify these roles e.g. training of junior doctors, research, etc.
- What proportion of your time is spent on your various roles?

**The next few questions will focus on your decision-making process when prescribing anticoagulants for adults requiring acute treatment (therefore patients who are 16 years and older).**

**Note: we are referring to the use of unfractionated heparin and low molecular weight heparin in inpatients only (therefore not the use of oral anticoagulants or anticoagulant choice for patients in the community).**

1. Can you please describe for me which indications you prescribe anticoagulation for?

Prompts:

- 1. ACS
  2. VTE (DVT/PE)
  3. Stroke
  4. CVST
  5. Other

1. Can you describe your opinion of the differences between unfractionated heparin (UFH) and Low Molecular Weight Heparin (LMWH)?
2. Do you have a preferred anticoagulant that you prescribe in the acute management phase? If so, what and why?
3. Can you please describe for me the decision-making process you go through prior to prescribing?
   1. unfractionated heparin
   2. low molecular weight heparin?
4. Considering your prescribing of anticoagulants:
   1. Are there specific factors that you consider during your decision-making process?

- Patient factors (e.g. age, renal function, bleeding risk)
- Diagnosis
  1. What role do various clinical decision support tools or resources play in your decision-making process?
- Which tools/ resources do you consider?
- How do you use/access these?
  1. Other than the patient, what else influences your decision-making process?
     Prompts:
- Cost (nurse administration, monitoring and pathology tests)
- Drug characteristics
- Departmental pressures
- Medicolegal concerns/risk avoidance
- Political factors

1. How do you assess bleeding risk? Are there any patient factors (history, comorbidities, planned interventions) that would make you consider the patient as a high bleeding risk?
2. Consider the following scenarios
   1. A 70-year-old female presents with an acute sub-massive PE after recent total knee replacement. Nil other medical history.

- What are your considerations for acute anticoagulant treatment?
- Are there any guidelines/decision support tools that you would refer to?
- What agent would you prescribe?
  1. A 65-year old male presents with a NSTEMI and is scheduled to undergo PCI in approximately 24 hours.
- What are your considerations for acute anticoagulant treatment?
- Are there any guidelines/decision support tools that you would refer to?
- What agent would you prescribe?
  1. A 55-year old female presents with AF and acute ischaemic stroke, unsuitable for thrombolysis.
- What are your considerations for acute anticoagulant treatment?
- Are there any guidelines/decision support tools that you would refer to?
- What agent would you prescribe?
  1. An 80-year-old male presents with CVST. History of CKD stage IV (eGFR 25). Nil other medical history.
- What are your considerations for acute anticoagulant treatment?
- Are there any guidelines/decision support tools that you would refer to?
- What agent would you prescribe?
