# Supplementary material for: Prescriber perceptions of the safety and efficacy of unfractionated heparin versus low molecular weight heparin in the acute treatment phase: a qualitative study
Source: J Pharm Policy Pract. 2024 Nov 19;17(1):2418367. doi: 10.1080/20523211.2024.2418367 (PMC11578413; doi:10.1080/20523211.2024.2418367)
Supplement: COREQ_checklist.docx [file JPPP_A_2418367_SM2592.docx]

Consolidated criteria for reporting qualitative studies (COREQ): 32-item checklist

**Developed from:**

Tong A, Sainsbury P, Craig J. Consolidated criteria for reporting qualitative research (COREQ): a 32-item checklist for interviews and focus groups. *International Journal for Quality in Health Care*. 2007. Volume 19, Number 6: pp. 349 – 357

**MANUSCRIPT TITLE: Prescriber perceptions of the safety and efficacy of unfractionated heparin versus low molecular weight heparin in the acute treatment phase: a qualitative study**

| **No. Item** | **Guide questions/description** | **Reported on Page #** |
| --- | --- | --- |
| **Domain 1: Research team and reﬂexivity** |  |  |
| *Personal Characteristics* |  |  |
| 1. Inter viewer/facilitator | Which author/s conducted the interview or focus group? | 5 |
| 2. Credentials | What were the researcher’s credentials? (E.g. PhD, MD) | 1 |
| 3. Occupation | What was their occupation at the time of the study? | 5 |
| 4. Gender | Was the researcher male or female? |  |
| 5. Experience and training | What experience or training did the researcher have? | 1,5 |
| *Relationship with participants* |  |  |
| 6. Relationship established | *Was a relationship established prior to study commencement?*  . | 5 |
| 7. Participant knowledge of the interviewer | *What did the participants know about the researcher? (e.g. personal goals, reasons for doing the research).* | 5 |
| 8. Interviewer characteristics | *What characteristics were reported about the interviewer/facilitator? (e.g. Bias, assumptions, reasons and interests in the research topic)* | 5 |
| **Domain 2: Study design** |  |  |
| *Theoretical framework* |  |  |
| 9. Methodological orientation and Theory | *What methodological orientation was stated to underpin the study? (e.g. grounded theory, discourse analysis, ethnography, phenomenology, content analysis).* | 4 |

| *Participant selection* |  |  |
| --- | --- | --- |
| 10. Sampling | *How were participants selected? (e.g. purposive, convenience, consecutive, snowball)* | 5 |
| 11. Method of approach | *How were participants approached? (e.g. face- to-face, telephone, mail, email)* | 5 |
| 12. Sample size | *How many participants were in the study?* | 6 |
| 13. Non-participation | *How many people refused to participate or dropped out? Reasons?* | 6 |
| *Setting* |  |  |
| 14. Setting of data collection | *Where was the data collected? (e.g. home, clinic, workplace)* | 4 |
| 15. Presence of non- participants | *Was anyone else present besides the participants and researchers?* | 4 |
| 16. Description of sample | *What are the important characteristics of the sample? (e.g. demographic data, date)* | 4 |
| *Data collection* |  |  |
| 17. Interview guide | *Were questions, prompts, guides provided by the authors? Was it pilot tested?* | 5 |
| 18. Repeat interviews | *Were repeat interviews carried out? If yes, how many?* | 6 |
| 19. Audio/visual recording | *Did the research use audio or visual recording to collect the data?* | 5 |
| 20. Field notes | *Were ﬁeld notes made during and/or after the interview or focus group?* | 5 |
| 21. Duration | *What was the duration of the inter views or focus group?* | 6 |
| 22. Data saturation | Was data saturation discussed? | 6 |
| 23. Transcripts returned | *Were transcripts returned to participants for comment and/or correction?* | 5 |
| **Domain 3: analysis and ﬁndings** |  |  |
| *Data analysis* |  |  |
| 24. Number of data coders | How many data coders coded the data? | 5 |

| 25. Description of the coding tree | *Did authors provide a description of the coding tree?* |  |
| --- | --- | --- |
| 26. Derivation of themes | *Were themes identiﬁed in advance or derived from the data?* | 5 |
| 27. Software | *What software, if applicable, was used to manage the data?* | 5 |
| 28. Participant checking | *Did participants provide feedback on the ﬁndings?* | NA |
| *Reporting* |  |  |
| 29. Quotations presented | *Were participant quotations presented to illustrate the themes/ﬁndings? Was each*  *quotation identiﬁed? (e.g. participant number)* | 9-17 |
| 30. Data and ﬁndings consistent | *Was there consistency between the data presented and the ﬁndings?* | 9-17 |
| 31. Clarity of major themes | *Were major themes clearly presented in the ﬁndings?* | 9-17 |
| 32. Clarity of minor themes | Is there a description of diverse cases or discussion of minor themes? | 9-17 |
